# Perceived effectiveness of the person–environment–occupation innovative teaching model in rehabilitation therapy students

**DOI:** 10.1186/s12909-024-06196-2

**Published:** 2024-10-21

**Authors:** Zhizhuo Wang, Peiyun Wu, Ming Li, Xiuyun Chen, Cheng Lin

**Affiliations:** Department of Rehabilitation Medicine, School of Health, Fujian Medical University, Fuzhou, 350122 China

**Keywords:** PEO innovative teaching model, Medical students, Rehabilitation therapy

## Abstract

**Background:**

Given the characteristics of the rehabilitation therapy specialty and the drawback of the traditional didactic teaching approach, we developed the innovative teaching approach from the students’ perspective using the person–environment–occupation (PEO) model. The new model was developed from the original PEO model to be applied in pedagogical contexts and tailored to the characteristics of an application-oriented specialty, such as rehabilitation therapy. The present study aimed to examine the perceived effectiveness of the PEO innovative teaching model applied to rehabilitation therapy students.

**Methods:**

A pre–post descriptive study was conducted to compare the perceived effectiveness of the PEO innovative teaching model for two consecutive student cohorts. A purposive sampling method was used to recruit students from two cohorts of junior medical students majoring in rehabilitation therapy in the spring semesters of 2020 and 2021. A questionnaire was developed to evaluate the perceived effectiveness of the PEO innovative teaching model. SPSS version 26.0 was used to perform data analysis.

**Results:**

A total of 112 students were included in the study and completed both pre- and post-tests. Of 112 received questionnaires, 101 were considered as valid questionnaires after the quality check (effective rate 90.18%). In the 2020 and 2021 cohorts, students rated the PEO innovative teaching model significantly higher than the didactic teaching approach in the following aspects: the teaching content is highly professional and valuable; the course is extensively expounded; the clinical reasoning is strengthened; problem-solving ability is developed; communication skills are improved; and teamwork skills are developed.

**Conclusion:**

The PEO innovative teaching model was perceived to be an effective teaching strategy to enhance students’ academic performance. In addition, three core skills including clinical reasoning, problem-solving ability, and communication skills were improved by the PEO innovative teaching model.

## Background

The World Health Organization (WHO) reported that the unexpected surge in the aging population and increased incidence of non-communicable diseases (NCDs) have increased markedly the demand for specialized rehabilitation therapists worldwide [1]. It is estimated that approximately one out of three persons currently has a need for rehabilitation services [1]. China, as one of the largest populations with > 1.4 billion, is experiencing a shortage of certificated professional rehabilitation therapists to provide value-based services [2]. According to the Healthy China 2030 report, the concept of early rehabilitation has been emphasized [3]. Healthy China 2030 is a comprehensive plan that focuses on improving the overall health of the Chinese population. Early rehabilitation can facilitate patients’ efforts to expediate functional restoration and improve their quality of life, thereby promoting the overall national health agenda. Likewise, the Rehabilitation 2030 initiative proposed by WHO also prioritizes the rehabilitation as a health strategy for optimizing the health and wellbeing of the global population [4]. Therefore, training more rehabilitation-related specialists is important.

In recent years, the number of colleges and universities where rehabilitation-therapy-related majors are accredited by the Chinese Ministry of Education has increased annually. In China, rehabilitation therapy at undergraduate educational level is an application-oriented specialty including physiotherapy, occupational therapy (OT), speech therapy, and Chinese traditional rehabilitation etc., aiming to train rehabilitation therapists with a variety of skills [5, 6]. Fujian Medical University set up a rehabilitation therapy specialty at undergraduate level since 2002. Medical students majoring in rehabilitation therapy are required to learn multiple rehabilitation-related skills, including physical agent modalities, OT techniques, and traditional Chinese acupuncture and massage. OT is a core component and compulsory course for medical students majoring in rehabilitation therapy. It has to be noted that occupations refer to the meaningful and purposeful activities that people need to, want to, and are expected to do in the course of OT [7].

Currently, didactic teaching has been universally used as the main strategy for all disciplines in university education in China [8]. However, it has to be acknowledged that this has focused more on teachers than students, and fails in an interactive/supportive learning context to enhance students’ academic performance. Students, as the recipients (customers) of teaching (services) offered by teachers (providers), significantly contribute to the instructional effectiveness of the teaching approach. As such, developing innovative teaching models from students’ perspectives can better understand students’ needs and expectations, as well as increase their learning initiative and motivation. Based upon the status quo aforementioned, we intended to develop a teaching approach from students’ perspectives using the person–environment–occupation (PEO) model (PEO innovative teaching model). The PEO model, developed by Law et al., is a practical model used by rehabilitation therapists, emphasizing that there are transactional relationships among the person, environment and occupation throughout the lifespan that can eventually affect occupational performance [9]. In the original PEO model, the person is assumed to be dynamic, motivated and to have ever-developing attributes; the environment is considered to be the non-static context where occupational performance occurs; and occupation is viewed as any activities or tasks done to accomplish a goal. The occupational performance is the product of their interactions. Practitioners and researchers can use the PEO model to conceptualize, plan, communicate and evaluate occupational performance interventions [10]. However, there is a lack of relevant studies researching the application of the PEO model in the pedagogical milieu. Therefore, the aim of this study was to examine the perceived effectiveness of the PEO innovative teaching model applied to rehabilitation therapy students.

## Methods

### Study design and population

A pre–post descriptive study was conducted to compare the perceived effectiveness of the PEO innovative teaching model for two consecutive student cohorts by using a purposive sampling method. The OT course is compulsory in the spring semester for junior medical students majoring in rehabilitation therapy. The inclusion criteria were as follows: (1) junior medical students majoring in rehabilitation therapy at Fujian Medical University in the spring semester of 2020 and 2021; and (2) students who registered for and completed the OT course. This study was granted exemption by the Fujian Medical University Biomedical Research Ethics Review Committee.

### Study intervention

**Fig. 1:**
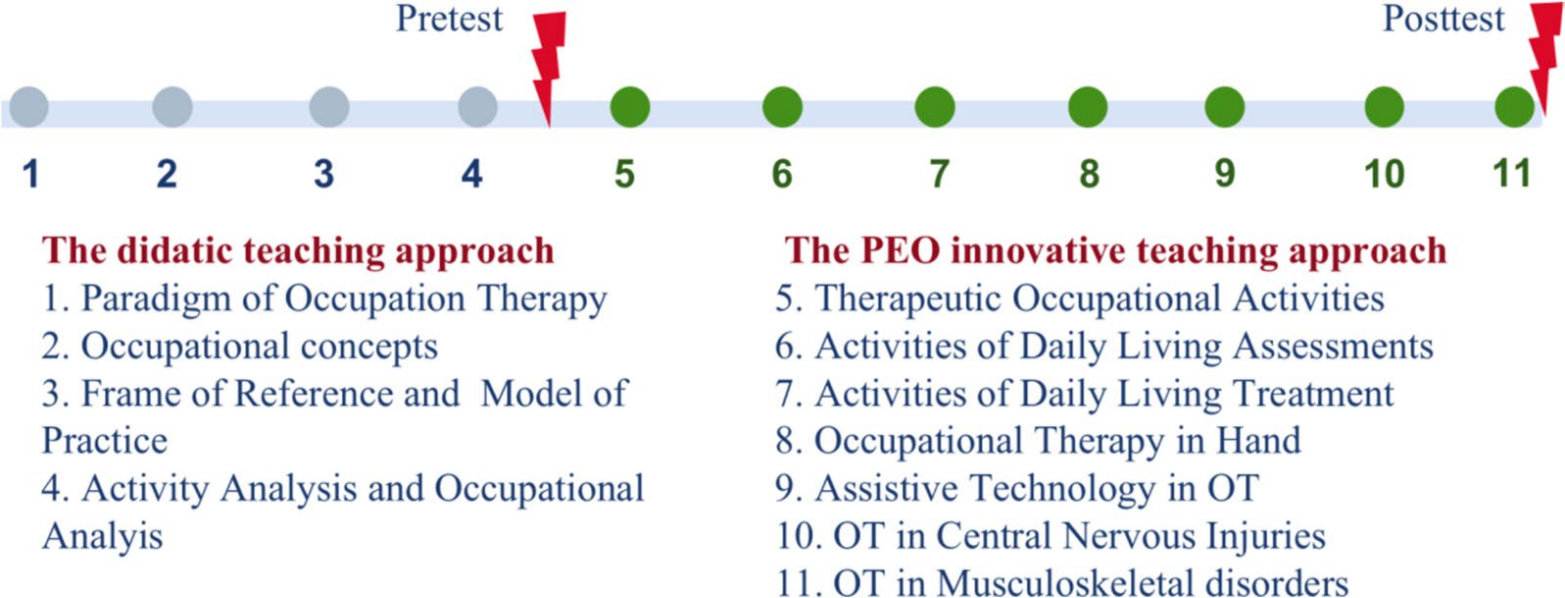
Timeline of data collection

The OT course involves 11 modules in total: the former four focus on fundamental theory (e.g., OT paradigm, occupational concepts, frame of reference, model of practice), and the latter seven on clinical practice (e.g., assessments and treatments) (Fig. 1). Due to the nature of teaching content, the didactic teaching approach is used in the former four modules, followed by the PEO innovative teaching model in the latter seven modules. The OT course is intended to give students a basic understanding of OT to supplement their medical skills, rather than providing them with anywhere near the foundation level of skills for this profession.

The PEO innovative teaching model was developed through a drafting phase and expert consultation. In the drafting phase, two students, two teaching assistants, and three teachers in the Department of Rehabilitation Medicine in Fujian Medical University gathered to brainstorm and outline the preliminary version of the PEO innovative teaching model. Specifically, three teachers drafted the original model based on the literature findings and teaching experiences. They held several virtual and in-person meetings with teaching assistants and students to seek inputs from their perspective. After several revisions, teachers, teaching assistants and students agreed on the preliminary version of the PEO innovative teaching model. In the phase of expert consultation, several experts were invited to revise and refine the draft. We used snowball sampling among faculty members in the Department of Rehabilitation Medicine to identify other eligible experts who could be invited to participate in the consultation. After three rounds of consultation, the final version of the PEO innovative teaching model was determined. The inclusion criteria for the invited experts were: (1) experts specialized in rehabilitation, physical or occupational therapy, including practitioners, teachers and researchers; (2) experts who had been engaged in pedagogical, clinical or research work for > 5 years; (3) experts with advanced academic positions, such as professors and associate professors; and (4) experts who were familiar with the PEO model.

**Fig. 2:**
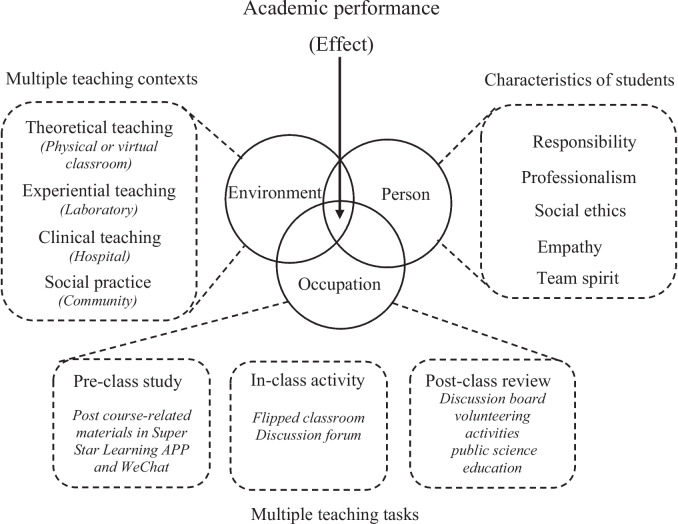
Diagrammatic framework of the PEO innovative teaching model

In the PEO innovative teaching model (Fig. 2), the “P” is redefined as characteristics of included students, such as students’ responsibility, professionalism, empathy, social ethics and team spirit. Taking the unit of assistive technology for example, students will be asked to draft a plan about how a wheelchair-bound patient with both legs amputated travels around the community, followed by implementing it in the simulated context, aiming to promote students’ critical reasoning, teamwork ability and professionalism. The “E” is reconsidered as creating multiple teaching environments in line with various teaching content. For example, instructors will use physical and/or virtual classrooms to impart theoretical knowledge (virtual classroom is used only if some students cannot attend classes in-person because of COVID-19). Experiential teaching is executed in provincial laboratory teaching centers (e.g., OT evaluation and treatment laboratory and virtual simulation laboratory) and provincial rehabilitation engineering research centers. Students will have the opportunity to be exposed to clinical contexts to strengthen clinical reasoning. The “O” is modified as multiple teaching tasks or activities. In pre-class study, the instructors will post some course-related materials in Super Star Learning APP and WeChat in order to familiarize students with the course content. During the class, the instructors will use cases and personal experiences to simplify some occupational complex concepts. Students will have opportunities to express themselves in the flipped classes and the forum discussions. In the post-class review, the discussion board will be open to encourage students to express their opinions. In addition, students will take advantage of their spare time to participate in volunteering activities and public science education, which enables them to be social contributors in the future. The degree of overlap between the three spheres represents students’ academic performance, and the closer the spheres overlap, the greater the effect of academic performance. Importantly, the PEO innovative teaching model cannot be effective without students’ participation. The overlap (academic performance) depends on the degree of students’ participation in the three spheres. To better understand the PEO innovative teaching model, we compared it with other innovative teaching methods [11–15] (Table 1).

**Table 1:** Comparison of PEO innovative teaching model with other innovative teaching methods

	Flipped classroom	Clinical practical teaching approach	Combined PBL and CBL method	Seminar teaching method	PEO innovative teaching model
Active discussion	YES	YES	YES	YES	YES
Group based work	YES	YES	YES	YES	YES
Case-based problem solving	YES	YES	YES	YES	YES
Scenario-based simulation	NO	YES	YES	NO	YES
Active participation in the learning experience	YES	NO	NO	YES	YES
Participation in clinical practice	NO	YES	NO	NO	YES
Addresses multiple learning styles	YES	NO	YES	NO	YES

*CBL* case-based learning, *PBL* problem based learning

### Instruments

Given the lack of standardized assessment tools available to evaluate the perceived effectiveness of the PEO innovative teaching model, a study-specific questionnaire was designed by literature review and consultations of two senior faculty members who have a background in creating evaluation instruments. Through three-round consultations, a consensus was reached on the final version of the questionnaire. The questionnaire uses a 10-point Likert scale ranging from 1 (extremely disagree) to 10 (extremely agree) with the midpoint of 5 (neutrally). The questionnaire involves 10 items: (1) the teaching content is highly professional and valuable; (2) the classroom atmosphere is engaging; (3) I am motivated by the course content; (4) the course is extensively expounded; (5) the clinical reasoning is strengthened; (6) critical thinking is activated by the course discussion; (7) problem-solving ability is developed; (8) communication skills are improved; (9) autonomous learning ability is fostered; and (10) teamwork skills are developed. The face validity of this questionnaire was confirmed by two senior faculty members with a background in creating evaluation instruments, and Cronbach’s α coefficient (0.978) was used to assess its reliability. If < 90% of the questionnaire items were filled out, and the speed of completion was less than one-third of the median length of this survey, the questionnaire was invalid.

### Data collection

Data were collected pre- and post-implementation of the PEO innovative teaching model. For the pre-test, after finishing the first four teaching modules, the anonymous questionnaires were distributed to students on the “Questionnaire Star” platform by a trained teaching assistant who was different from the teaching assistant in the development of the PEO innovative teaching model. At the end of the latter seven teaching modules, these students were asked to fill out the questionnaires as the post-test. The timeline of data collection is illustrated in Fig. 1. Before the pre-test, students were informed that engagement in the survey was completely voluntary, and any personal identifiable information was removed or masked (the questionnaire was totally anonymous), and that the final grade obtained would not be affected by participating or not. Students were required to fill out questionnaires that could be accessed only once in order to ensure the quality of the survey. Only when 90% of items in the questionnaire were answered could it be considered a valid response.

### Statistical analysis

Data analysis was performed using SPSS version 26.0 (Chicago, IL, USA). The normality was examined by the Shapiro–Wilk test, indicating that the data were not normally distributed. Descriptive data were reported as frequency and percentage (%). Continuous data were expressed as medians (1st and 3rd quartiles). We used the Mann–Whitney U test to analyze the pre- and post-test data. The median imputation was used to handle the missing data. *P* < 0.05 was considered as statistically significant.

## Results

### Basic characteristics of included students

A total of 112 students were included in the study and completed both pre- and post-tests as required. Table 2 provided a summary of the demographics of included students. There were no significant differences between students in the 2020 and 2021 cohorts.

**Table 2:** Basic characteristics of the students in the 2020 and 2021 cohorts

	Cohorts			
	2020 (*N* = 55)		2021 (*N* = 57)	
Sex Number	Male	Female	Male	Female
	18	37	18	39
Age (yr) Number	21	22	21	22
	30	25	28	29

Perceived effectiveness of the PEO innovative teaching model.

A total of 112 questionnaires were distributed and all were returned, but only 101 were considered as valid after the quality check (effective rate was 90.18%).

In both cohorts, students rated the PEO innovative teaching model significantly higher than the didactic teaching approach in the following aspects: teaching content was highly professional and valuable; the course was extensively expounded; clinical reasoning was strengthened; problem-solving ability was developed; communication skills were improved; and teamwork skills were developed (Table 3).

**Table 3:** Comparison before and after implementation of the PEO innovative teaching model

Items	Pre-test		Post-test		*P* value	
	2020	2021	2020	2021	2020	2021
Teaching content highly professional and valuable	8.17 (7.42, 9.50)	8.00 (6.42, 9.42)	9.84 (9.08, 10.0)	10.0 (9.08, 10.0)	0.017	0.014
Classroom atmosphere is engaging	9.17 (8.18, 9.67)	8.67 (7.33, 10.0)	10.0 (8.75, 10.0)	10.0 (8.17, 10.0)	0.129	0.244
I am motivated by the course content	9.17 (8.08, 10.0)	9.33 (8.0, 10.0)	10.0 (8.75, 10.0)	9.50 (8.25, 10.0)	0.362	0.759
Course is extensively expounded	8.67 (8.0, 10.0)	7.67 (7.33, 8.67)	10.0 (9.25, 10.0)	9.50 (8.75, 10.0)	0.033	0.001
Clinical reasoning is strengthened	7.84 (6.67, 8.59)	7.67 (6.08, 8.33)	10.0 (8.58, 10.0)	9.84 (9.08, 10.0)	0.001	0.001
Critical thinking is activated by the course discussion	8.33 (8.0, 10.0)	8.84 (8.08, 9.83)	9.84 (8.84, 10.0)	10.0 (8.75, 10.0)	0.149	0.131
Problem-solving ability is developed	7.33 (5.92, 8.33)	8.00 (6.58, 9.25)	10.0 (8.67, 10.0)	9.84 (9.0, 10.0)	< 0.001	0.005
Communication skills are improved	7.50 (5.67, 7.92)	7.50 (5.67, 8.0)	9.50 (8.33, 10.0)	9.84 (9.0, 10.0)	< 0.001	< 0.001
Autonomous learning ability is fostered	9.34 (7.75, 10.0)	9.50 (8.42, 10.0)	10.0 (8.58, 10.0)	9.67 (9.0, 10.0)	0.438	0.521
Teamwork skills are developed	7.50 (6.17, 8.0)	7.17 (6.08, 8.75)	10.0 (9.25, 10.0)	10.0 (9.33, 10.0)	< 0.001	0.003

## Discussion

Although the PEO model has been universally used in the practical and research-related domains, there has been little evidence of its application in pedagogical contexts. To our knowledge, this is the first study to apply the PEO model in teaching contexts and to examine its perceived effectiveness. Compared with the didactic teaching approach, students rated the PEO innovative teaching model higher in several aspects. To better understand the PEO innovative teaching model, we need to decipher the original and adapted components of the PEO model and their interactions. The person, environment and occupation correspond to the student, classroom and teaching tasks, respectively. Based on this assumption, we redefined the three components of PEO model for application to the educational milieu. The PEO model has also been used as an analytic tool or framework in multiple clinical scenarios, such as pediatrics [16], cardiac rehabilitation [17] and mental health [18].

Our findings suggested that students rated the PEO innovative teaching model highly. Specifically, three core skills were improved in the process of learning, including clinical reasoning, problem-solving ability and communication skills, which echoed previous studies [19–21]. For example, Murphy and Radloff revealed that case-based learning could influence the clinical reasoning of OT students, minimizing the reliance on rules-based thinking and external feedback in the reasoning process [22]. Park et al. pointed out that extended exposure to simulation-based training could intensify their learning motivation and improve their problem-solving abilities [23]. Concerning communication skills, researchers have found that students engaged in the interprofessional simulation had significant improvements in communication and teamwork skills [24].

The difference between the PEO innovative teaching model and the didactic teaching approach was not significant for the item “I am motivated by the course content”. Motivation, defined as a student’s desire to achieve excellence, is an important factor relating to academic performance and scholarly achievement, intention to drop out, and absenteeism [25, 26]. As shown in the evidence, motivation can be affected by the curriculum structures and the impact may be individualized [27]. The study of Li et al. supported this viewpoint that students in the PEO model experiential teaching group had a higher level of motivation to participate in classes [28]. The PEO model in that study was different from our PEO innovative teaching model in that they only used the PEO model as an analytic tool in a specific clinical scenario. It is noteworthy that intrinsic motivation can be bolstered through virtual gamification, indicating that gamification features may be integrated into course content to increase students’ learning motivation [29]. Lastly and importantly, it is recommended that educators can integrate some motivational elements into the PEO innovative teaching model so as to promote students’ motivation to participate.

The strength of this study was that the PEO innovative teaching model considers activities that students undertake outside of universities, such as volunteering and public science education. However, there were some limitations to our study. Firstly, the questionnaire used to assess the effectiveness of the PEO innovative teaching model was developed by rehabilitation therapy experts. Although we examined the internal consistency of the developed questionnaire (Cronbach’s α 0.978) based upon the data collected, the psychometrics of this questionnaire need to be further explored. Secondly, due to the educational justice, we did not divide students into the PEO innovative teaching model group and the didactic teaching approach group. Alternatively, students filled out questionnaires pre- and post-implementation of the PEO innovative teaching model. Lastly, the study was conducted at Fujian Medical University and participants were recruited using a purposive sampling strategy, which may influence our ability to generalize our findings worldwide.

## Conclusion

The PEO innovative teaching model was perceived to be an effective teaching strategy to enhance students’ academic performance. In addition, three core skills including clinical reasoning, problem-solving ability, and communication skills were improved by the PEO innovative teaching model. However, there were no significant differences between the PEO innovative teaching model and didactic teaching approach for the following aspects: I am motivated by the course content; the classroom atmosphere is engaging; critical thinking is activated by the course discussion; and autonomous learning ability is fostered. Thus, the next step is to refine the PEO innovative teaching model and test its effectiveness in other Chinese universities to promote its generalization.

## Data Availability

The datasets used and/or analyzed in this study are available from the corresponding author or first author on reasonable request.

## Abbreviations

PEO: Person-environment-occupation
OT: Occupational therapy
PBL: Problem based learning
CBL: Case-based learning
